# SurvivalGWAS_SV: software for the analysis of genome-wide association studies of imputed genotypes with “time-to-event” outcomes

**DOI:** 10.1186/s12859-017-1683-z

**Published:** 2017-05-19

**Authors:** Hamzah Syed, Andrea L. Jorgensen, Andrew P. Morris

**Affiliations:** 1 Department of Biostatistics, University of Liverpool, Liverpool, UK; 2 Department of Molecular and Clinical Pharmacology, University of Liverpool, Liverpool, UK

**Keywords:** Genome-wide association study, Pharmacogenetics, Time to event, Cox proportional hazards, Weibull, Survival analysis, SNP-covariate interaction

## Abstract

**Background:**

Analysis of genome-wide association studies (GWAS) with “time to event” outcomes have become increasingly popular, predominantly in the context of pharmacogenetics, where the survival endpoint could be death, disease remission or the occurrence of an adverse drug reaction. However, methodology and software that can efficiently handle the scale and complexity of genetic data from GWAS with time to event outcomes has not been extensively developed.

**Results:**

SurvivalGWAS_SV is an easy to use software implemented using C# and run on Linux, Mac OS X & Windows operating systems. SurvivalGWAS_SV is able to handle large scale genome-wide data, allowing for imputed genotypes by modelling time to event outcomes under a dosage model. Either a Cox proportional hazards or Weibull regression model is used for analysis. The software can adjust for multiple covariates and incorporate SNP-covariate interaction effects.

**Conclusions:**

We introduce a new console application analysis tool for the analysis of GWAS with time to event outcomes. SurvivalGWAS_SV is compatible with high performance parallel computing clusters, thereby allowing efficient and effective analysis of large scale GWAS datasets, without incurring memory issues. With its particular relevance to pharmacogenetic GWAS, SurvivalGWAS_SV will aid in the identification of genetic biomarkers of patient response to treatment, with the ultimate goal of personalising therapeutic intervention for an array of diseases.

## Background

Genome-wide association studies (GWAS) have revolutionised our understanding of the genetic basis of a wide variety of complex human traits and diseases. GWAS are designed to detect associations between single nucleotide polymorphisms (SNPs) across the entire genome and outcome. The focus of most GWAS have been binary phenotypes or quantitative traits, for which proficient software tools for analysis have been developed, such as SNPTEST [1] and PLINK [2].

“Time-to-event” outcomes have become increasingly relevant, particularly in the context of pharmacogenetic studies, where the outcome of interest could be based on overall survival [3], time to remission [4] or progression-free survival [5] after treatment/therapy intervention. The traditional approach to the analysis of time to event data is through survival modelling, and the underlying models used are the same when applied to genetic association studies. However, the challenge arises from the scale and complexity of genetic data, and the need to incorporate a range of analytical models, which require computationally efficient software. Currently, there is a paucity of such powerful tools for survival analysis of GWAS.

There are many recent GWAS published with a focus on survival outcomes such as He et al. [6], Phipps et al. [7], Johnson et al. [8] and Wu et al. [9]. In these studies, genome-wide time to event analyses were conducted using standard statistical software, such as R or SAS, which are limited by memory and not easily amenable to high-performance computing (HPC) solutions to improve efficiency. Programs such as ProbABEL [10] exist for this type of analysis, but are limited to the use of only the Cox proportional hazards model and also do not allow exploration of SNP-covariate interaction effects. This is a particularly important feature for the analysis of pharmacogenetic data, where it is often desirable to test for drug or dose interactions with SNPs.

We have developed the software tool SurvivalGWAS_SV, which has addressed these challenges, and currently employs a single SNP analysis approach using two commonly used analysis models. Key features include: (i) compatibility with widely used programs such as IMPUTE [11], thereby directly accommodating imputed data without the need for file conversion; (ii) a range of survival analysis methods are available with the foundation in place for implementing extensions; (iii) options for testing SNP-covariate interactions, showing overall and individual test of association *p*-values; and (iv) compatibility with high performance parallel computing clusters.

SurvivalGWAS_SV is the second program to be released under the SurvivalGWAS Suite, which also includes the complementary power calculator “SurvivalGWAS_Power” [12].

## Implementation

### User interface

SurvivalGWAS_SV is a console application utilising command line inputs. The software is run from a command prompt terminal, compatible with Linux, Windows and Mac OS X. The program requires little interaction from the user since a script of commands can be submitted to the program. This is useful for the analysis of large data files: the user can specify “batches” of the data file to analyse in parallel using multiple computer nodes, where each core can run a different part of the analysis. The program requires Mono [13] to run the software on Linux and Mac OS X, but this does not compromise speed or efficiency.

### Inputs

SurvivalGWAS_SV is set up in a very simplistic way. Firstly, the user is required to specify the two data files that will be read into the program. This must be a genotype file (.gen or.impute) or a variant call format (VCF) text file that contains the SNP genotype probabilities (imputed or non-imputed), and a sample file (.sample) that contains all the covariate, survival time and censoring indicator information for each individual. The software supports VCF files containing the SNP genotype probabilities, dosages and/or hard genotype calls. In some circumstances, the user would have the genotype files compressed, either in a.zip or.gz file format, both of which can be read into the software directly. Secondly, the user specifies details about terms to include in their analysis model, such as covariates and/or interaction, whilst also specifying the censoring indicator and observed survival time. Thirdly, the user must specify the range of SNPs to be analysed, to enable efficient parallel computing. Lastly, the user must enter the chosen analytical method to use and the name of the file for which the analysis output will be saved. If the user is analysing covariates within the model, but does not require summary statistics for the covariates to be included in the output file, an option is available for only printing the results for the SNP or interaction effects. This is helpful when creating graphical summaries, such as Manhattan plots, using other programs. Table 1 gives a brief description of all the available commands.

**Table 1:** List of commands available in the software and their corresponding usage description

Command	Description
-gf=	This specifies the genotype file. Typically .gen, .impute, .gen.gz.
-sf=	This specifies the sample file (.sample).
-t=	This specifies the time to event (column heading name) in the sample file.
-c=	This specifies the censoring indicator/outcome in the sample file.
-cov=	This specifies the covariates to adjust for in the model. Each one separated by a comma (,). Categorical factors need to be converted to binary as software only assumes continuous or binary covariates.
-lstart=	This specifies the line in the genotype file at which the start position of analysis will occur. Used to break large files into small batches for parallel computing.
-lstop=	This specifies the line in the genotype file at which the end position of analysis will occur. Typically the number of lines is equal to the number of SNPs in the file.
-sp=	The start position (in base pairs) on the chromosome. Still need to specify the number of lines in the file using -lstart & -lstop commands. <optional>
-ep=	The stop position (in base pairs) on the chromosome. <optional>
-chr=	This specifies the chromosome number to be output in the text file.
-p=	Enter “onlysnp” if only the results from the SNP analysis are to be output and “onlyint” if only the results from the SNP-covariate interaction analysis are to be output. <optional>
-m=	This specifies the choice of method for analysis. This is either “cox” for the Cox proportional hazards model or “weibull” for the parametric Weibull regression model.
-o=	This specifies the name of the file for output to be saved in. e.g., name.txt
-help	Outputs a full list of commands and usage help.

### Conversion & validation

Before the data can be analysed, a number of conversions and quality control measures must be performed by the software. When the genotype file is read in, one SNP at a time, either directly typed or imputed, SurvivalGWAS_SV will convert the genotype probabilities for each subject into a “dosage” under an additive model for the minor allele. This enables appropriate analysis for imputed SNP data by taking account of the uncertainty in the imputation process. The dosage model is given by ${S}_i={p}_{i1}+2{p}_{i2}$, where ${p}_{i1}$ and ${p}_{i2}$ are the probabilities that subject $i$ carries 1 or 2 minor alleles, respectively, at the SNP.

SurvivalGWAS_SV throws exemptions whenever the user has specified an incorrect command or states a header that cannot be found in the data files. In such an event, the program will exit the application and will require re-submission of the task. The program also handles missing values within the .sample file. If a subject has missing values (in the form of “NA”) for survival time, censoring indicator or a covariate used in the model then the subject is removed from the analysis with their corresponding SNP information.

### Analysis

Analysis can be carried out using one of two methods: (i) a Cox proportional hazards model; or (ii) a parametric Weibull regression model. Both methods have their advantages under different scenarios. More details about power and choice of method can be found in Syed et al. [14]. Software for performing power calculations under a range of pharmacogenetic time to event scenarios is also available from Syed et al. [12].

The Cox proportional hazards model is widely considered the ‘standard’ approach when modelling time to event outcomes. It is a semi-parametric model where the hazard ratio takes a parametric form in terms of the regression coefficients, but the baseline hazard is unspecified. A disadvantage of this model is that the distribution of survival times is unknown. In cases where the proportional hazards assumption is not valid, other analysis models or extensions to the Cox-regression model should be considered.

The Weibull regression model is a parametric survival model with completely specified hazard and survivor functions. The Weibull model is beneficial when the hazard ratio is not proportional over time or the data have an accelerated failure time feature. For more information on the estimation of the Weibull regression model parameters please refer to Syed et al. [12].

### Output

The output from the analysis is saved in a text file, the name of which is specified by the user. Each individual parameter analysed is recorded in a list under a header row that specifies the values in each column. It includes the variable name (can be the SNP ID, covariate or interaction name), rs ID, chromosome number, base-pair position, effect and non-effect alleles, coefficient value for each variable analysed, along with its hazard ratio, standard error, confidence intervals (only for Cox proportional hazards) and corresponding *p*-value (Wald test for Cox model and a score test for the Weibull model). The Weibull regression model output will also comprise of a row for the intercept and shape parameter. There is also output for the likelihood ratio test of the overall model, effect allele frequency (the frequency at which the most common allele occurs within a population), minor allele frequency (MAF) and the IMPUTE info measure of imputation quality [1].

### Example commands

Assuming all data files and software are in the same folder, the command line in a Linux terminal for the analysis of 10000 SNPs and 2 additional covariates using a Cox proportional hazards model is as follows:

mono SurvivalGWAS_SV.exe -gf=data.gen -sf=data.sample -t=event_times -c=censoring -cov=covariate1,covariate2 -chr=1 -lstart=0 -lstop=10000 -m=cox -p=onlysnp -o=output.txt

Each command is separated by a space. The user can specify the exact location of the data files and where the output file will be saved. e.g., /DIRECTORY/DATA/output.txt

An example of a shell script (.sh) to distribute the analyses between 10 computer cores within a Linux cluster, using a sun grid engine batch system is as follows:

#!/bin/bash

#$ -o stdout

#$ -e stderr

DIRECTORY=/SurvivalGWAS_SV #Location of software and data

str1=0 #Start position in genotype file

str=10000 #Number of SNPs/lines in genotype file

no_of_jobs=10 #Number of cores

inc=`expr \($str - $str1 \) \/ $no_of_jobs` #Increment

#SGE_TASK_ID takes values 1:no_of_jobs

nstart=`expr \($SGE_TASK_ID - 1 \) \* $inc’

nstop=`expr $nstart + $inc – 1`

mono $DIRECTORY/SurvivalGWAS_SV.exe –gf=$DIRECTORY/data.gen –sf=$DIRECTORY/data.sample -t=event_times -c=censoring -cov=covariate1,covariate2 -chr=1 -lstart=$nstart -lstop=$nstop -m=cox -p=onlysnp -o=$DIRECTORY/output${SGE_TASK_ID}.txt

## Results and discussion

To evaluate the performance of SurvivalGWAS_SV, we simulated genotype data using the software HAPGEN2 [15], based on European ancestry individuals from the HapMap3 [16] reference panel. Approximately 1.5 million SNPs were simulated across 22 chromosomes for 1000 patients. We then selected one SNP (rs12425539) on chromosome 12 as the causal variant, which we used to generate time to event data. We generated the time to event data using the power calculator software “SurvivalGWAS_Power”, which simulated the survival time and censoring indicator for each individual for this single replicate of genotype data at the causal SNP. A treatment covariate (binary) was also simulated for each patient using a binomial distribution. The active treatment and the placebo were divided evenly (1:1) between the 1000 patients. Four datasets were simulated with censoring occurring randomly for approximately 20% of the sample: (i) proportional hazards data with a significant SNP effect only; (ii) proportional hazards data with significant SNP, treatment and interaction effect; (iii) accelerated failure time data with a significant SNP effect only; and (iv) accelerated failure time data with significant SNP, treatment and interaction effect. Datasets (i) and (ii) were analysed using the Cox proportional hazards model, whereas datasets (iii) and (iv) were analysed using the Weibull regression model. Only the SNP term was included in the analysis models for analysing datasets (i) and (iii). Datasets (ii) and (iv), included SNP, treatment and interaction terms within the analysis models. After analysis, the number of SNPs was reduced by removing SNPs with a MAF < 0.01. This was to remove rare variants for which there is minimal power to detect association, and a standard procedure in GWAS quality control.

**Fig. 1:**
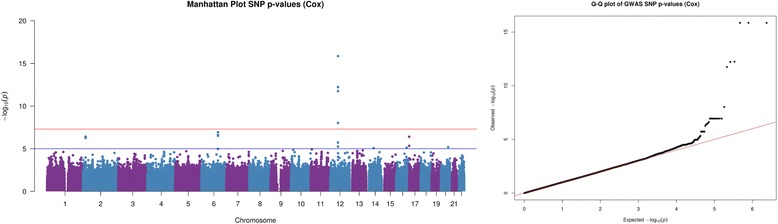
Graphical representation from proportional hazards data SNP analysis. Graphical output from simulation study. (*Left*) Manhattan plot of Cox proportional hazards analysis SNP *p*-values & (*Right*) Cox proportional hazards analysis QQ-plot

**Fig. 2:**
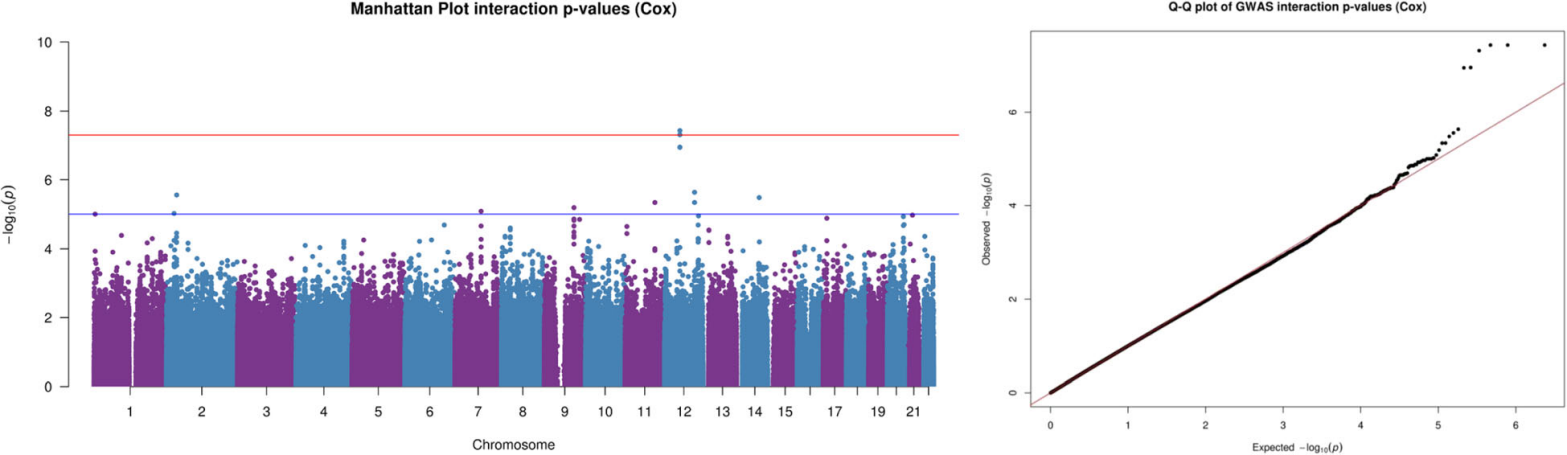
Graphical representation from proportional hazards data SNP-Treatment interaction analysis. Graphical output from simulation study. (*Left*) Manhattan plot of Cox proportional hazards analysis interaction *p*-values & (*Right*) Cox proportional hazards analysis QQ-plot

Figure 1 presents the results from the Cox proportional hazards model depicted by Manhattan and QQ-plots for dataset (i). The Cox proportional hazards analysis was able to detect the causal SNP association, identifying SNPs to be genome-wide significant (*p* < 5×10^−8^) in the data simulated using the proportional hazards model. The same can also be said when considering Fig. 2, which depicts the interaction analysis (SNP-treatment interaction *p*-values) for dataset (ii), simulated using the proportional hazards model.

**Fig. 3:**
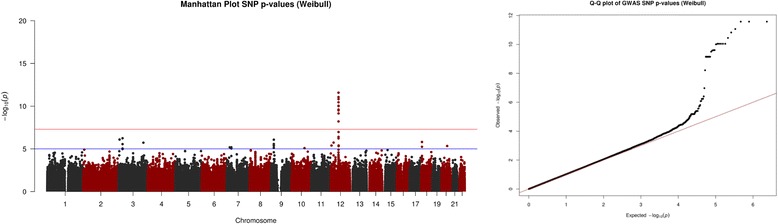
Graphical representation from accelerated failure time data SNP analysis. Graphical output from simulation study. (*Left*) Manhattan plot of Weibull regression analysis SNP *p*-values & (*Right*) Weibull regression analysis QQ-plot

**Fig. 4:**
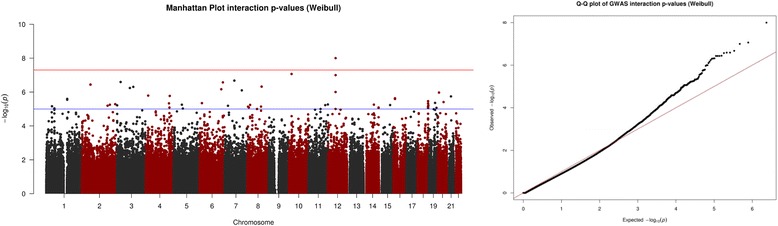
Graphical representation from accelerated failure time data SNP-Treatment interaction analysis. Graphical output from simulation study. (*Left*) Manhattan plot of Weibull regression analysis interaction *p*-values & (*Right*) Weibull regression analysis QQ-plot

Figures 3 and 4 represent the results from analysing the datasets simulated using the accelerated failure time assumption. Figure 3 shows us that the Weibull regression analysis identified the association between the causal SNP and time to event outcome. Figure 4 indicates that the Weibull regression model was able to detect the interaction effect in dataset (iv).

The entire analysis was run using 8 computer nodes (64 cores). Each node consisted of a HP Proliant DL170h G6 server, 2 Intel Xeon(R) E5520 2.27GHz quad-core CPUs, 36 GB memory and 1 TB of local storage. Running the single SNP analysis of 1.5 million SNPs across 22 chromosomes for 1000 individuals with no additional covariates took ~6 h to complete using the Cox proportional hazards model and ~5 h to complete using the Weibull regression model. The more covariates added to the analysis and/or the addition of an interaction, the longer the computational runtime. Each additional covariate took approximately an extra 0.275 s for each individual SNP analysed. The Weibull regression analysis runtime varies greatly; this is due to the convergence criteria of the Newton-Raphson method used for estimation of all parameters [12]. Runtime is also dependent on missing values within the sample file and whether or not the genotype file is compressed. Ultimately, cluster specifications and size of data files are the most influential factors affecting the speed of the software.

## Conclusion

SurvivalGWAS_SV is the first analytics software capable of applying a range of survival analysis methods to genome-wide data, with appropriate handling of imputed genotypes. The software can be applied to large-scale GWAS datasets efficiently and effectively, without incurring memory issues.

Survival analysis methodology is evolving quickly, with the majority of researchers implementing new methods within the R statistical environment. Future versions of SurvivalGWAS_SV will employ more complex analysis techniques and extensions to account for more complex survival models such as competing risks, whilst integrating with R to allow for the software to update methodological changes faster.

SurvivalGWAS_SV will ultimately enable discovery of genetic biomarkers of patient response to treatment for a range of complex human diseases, and will offer opportunities for patient stratification according to predicted benefit or risk of treatment, allowing personalisation of therapeutic intervention.

## Abbreviations

GWAS: Genome-wide association studies
HPC: High-performance computing
MAF: Minor allele frequency
SNP: Single nucleotide polymorphism
VCF: Variant call format
